# d-Lactic acid production from agricultural residue*s* by membrane integrated continuous fermentation coupled with B vitamin supplementation

**DOI:** 10.1186/s13068-022-02124-y

**Published:** 2022-03-04

**Authors:** Kedong Ma, Yubo Cui, Ke Zhao, Yuxuan Yang, Yidan Wang, Guoquan Hu, Mingxiong He

**Affiliations:** 1 Key Laboratory of Biotechnology and Bioresources Utilization, Ministry of Education, Dalian Minzu University, Dalian, 116600 People’s Republic of China; 2 College of Environment and Resources, Dalian Minzu University, Dalian, 116600 People’s Republic of China; 3 Key Laboratory of Development and Application of Rural Renewable Energy, Ministry of Agriculture, Biomass Energy Technology Research Centre, Biogas Institute of Ministry of Agriculture, Chengdu, 610041 People’s Republic of China; 4 Key Laboratory of Songliao Aquatic Environment, Ministry of Education, Jilin Jianzhu University, Changchun, 130118 People’s Republic of China

**Keywords:** d-Lactic acid, *Lactobacillus delbrueckii*, Lignocellulosic biomass, B vitamin supplementation, Cell-recycling continuous fermentation

## Abstract

**Background:**

d-Lactic acid played an important role in the establishment of PLA as a substitute for petrochemical plastics. But, so far, the d-lactic acid production was limited in only pilot scale, which was definitely unable to meet the fast growing market demand. To achieve industrial scale d-lactic acid production, the cost-associated problems such as high-cost feedstock, expensive nutrient sources and fermentation technology need to be resolved to establish an economical fermentation process.

**Results:**

In the present study, the combined effect of B vitamin supplementation and membrane integrated continuous fermentation on d-lactic acid production from agricultural lignocellulosic biomass by *Lactobacillus delbrueckii* was investigated. The results indicated the specific addition of vitamins B_1_, B_2_, B_3_ and B_5_ (VB_1_, VB_2_, VB_3_ and VB_5_) could reduce the yeast extract (YE) addition from 10 to 3 g/l without obvious influence on fermentation efficiency. By employing cell recycling system in 350 h continuous fermentation with B vitamin supplementation, YE addition was further reduced to 0.5 g/l, which resulted in nutrient source cost reduction of 86%. A maximum d-lactate productivity of 18.56 g/l/h and optical purity of 99.5% were achieved and higher than most recent reports.

**Conclusion:**

These findings suggested the novel fermentation strategy proposed could effectively reduce the production cost and improve fermentation efficiency, thus exhibiting great potential in promoting industrial scale d-lactic acid production from lignocellulosic biomass.

**Graphical Abstract:**

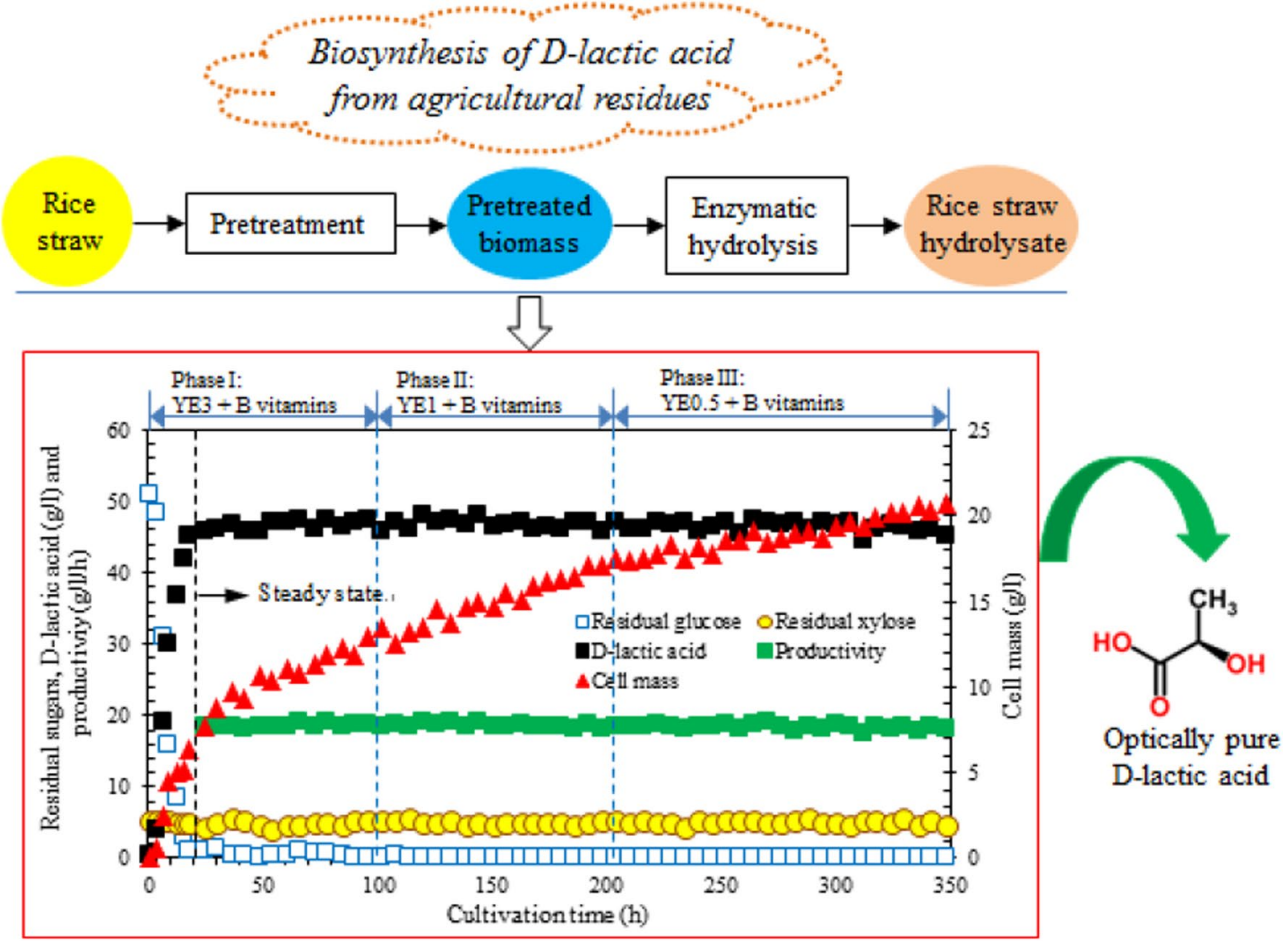

**Supplementary Information:**

The online version contains supplementary material available at 10.1186/s13068-022-02124-y.

## Highlights

- High d-lactic acid productivity is achieved by *L. delbrueckii* from rice straw.
- B vitamins are satisfied substitute of yeast extract for d-lactic acid fermentation.
- A process of membrane-integrated continuous fermentation with B vitamin is developed.
- High fermentation efficiency is achieved by the novel fermentation process.

**Supplementary Information:**

The online version contains supplementary material available at 10.1186/s13068-022-02124-y.

## Background

Lactic acid, an important hydroxycarboxylic acid that presents the two optical isomer forms in terms of l- and d-isomer, has traditionally applied in the chemical, pharmaceutical, cosmetic, and food industries [1–3]. In recent decades, optically pure lactic acid (l- or d-isomer) has attracted worldwide attention as a building block for the polylactic acid (PLA: PLLA and PDLA) manufacture [4]. PLA is one of the most eco-friendly biodegradable polymers and is generally recognized as a green alternative to traditional petroleum-derived plastics [5, 6]. However, the application of PLLA and PDLA is still limited due to their mechanical and thermal properties [7]. A polymer blend of enantiomerically pure PLLA and PDLA form a stable stereocomplex (Sc-PLA) which exhibit great advantages in mechanical strength, thermochemical properties and hydrolysis resistance compare to single-enantiomer polymers [8]. Sc-PLA provide new insight into d-lactic acid application, and thus trigger increasing interest in high enantiomeric purity d-lactic acid [9, 10]. Pure d- or l-isomers of lactic acid can be obtained through biotechnological microbial fermentation [11, 12]. Nowadays, the fermentative production of high-purity l-isomers had been established on an industrial scale, but research related to enantiomeric pure d-isomer production remains in the laboratory stage [13]. To meet the rapidly growing market requirement, a cost-effective industrial process for enantiomeric pure d-isomer production via microbial fermentation should be urgently developed and implemented [14, 15]. As well known, the production cost of pure d-isomer is a serious obstacle hindering their industrial application. Among the factors that contributed to the higher production cost of lactic acid, raw materials, nutrient sources and production processes were considered to be the major factors [16, 17]. To overcome these problems, much effort has been made to look for affordable substrates from various renewable resources over the past decades, and the studies revealed that inexpensive renewable raw feedstocks [18, 19], such as lignocellulosic biomass (2nd-generation (2G) feedstocks) from agricultural residues and agro-industrial or forestry sources, are the promising fermentative substrates for reducing the cost of lactic acid production [20–23].

Most lactic acid-producing bacteria (LAB) are heterotrophic and generally require many growth factors including nucleotides, vitamins, amino acids, peptides and fatty acids, for cell growth and biosynthetic capabilities [24, 25]. Yeast extract (YE), an effective complex nutrient source, is still the nutrient source of choice in many l-lactic acid fermentation processes, but its high price impedes its usage in industrial-scale production. Based on the economic analysis of lactate production, the YE cost could constitute up to 38% of total operational costs, suggesting the requirement for an economic alternative [26]. Up to now, the substitution of YE by cheap nutrient sources has been intensively studied in many microbial fermentation processes. Some agro-industrial wastes such as cottonseed [27], peanut meal [28] and soybean meal [29] have been well assessed as YE substitutes during D-lactate fermentation. By the application of those substitutes, the nutrient cost could be reduced to some extent, whereas some issues e.g. increasing impurities in fermented broth due to a great deal of nutrient source supplementation, might cause serious problems in downstream processing and subsequently give negative influence on industrial-scale d-lactic acid production.

B vitamins are a group of trace organic compounds that play an important role in microorganism growth and metabolism. Some attempts have been made to replace YE with B vitamins in lactic acid fermentation. Yoo et al. [30] reported the joint effect of soybean hydrolysate (SH) and B vitamins on lactic acid production. The addition of five B vitamins resulted in complete glucose consumption with 5 g/l YE, half the usual level. Kwon et al. [30] successfully replaced YE with SH supplemented with seven B vitamins, achieving a productivity of 2.27 g/l/h with lactic acid yield of 0.92 g/g. Nancib et al. [31] proved the possibility of partially substituting YE with inexpensive nitrogen sources and B vitamins in the study of lactate fermentation from date juice. However, the facts in these studies, such as the relatively low fermentation efficiency and large proportion of YE in mixed nutrient sources, are still a focus for further research.

The fermentation process is another important factor affecting the efficiency of biotechnological d-isomer production [32]. Comparative studies of various operation processes were performed including batch, fed-batch, repeated-batch (semi-continuous) and continuous fermentation by a wide range of homofermentative D-LAB [9]. Continuous techniques have advantage of reducing process costs through constantly feeding nutrients to keep their optimal concentration for cell growth [33, 34]. Therefore, the productivity and yield of lactate obtained is significantly higher than other fermentation modes [9]. However, some inherent defects in conventional continuous techniques such as the efflux of non-utilized substrates and cell loss still need to be resolved [1]. The efficient membrane-integrated continuous fermentation (MICF) system has been developed and assessed in several experimental studies. By coupling specific membrane with bioreactor, the problem of cell loss is effectively overcome. And by employing optimal dilution rate, the issue of membrane fouling, causes by increase in cell density could be efficiently resolved, consequently facilitate the stable long-term continuous fermentation. Different microfilter elements have been used as membrane in these studies. The results declare that MICF system could achieve the necessary cell densities and high d-lactic acid productivity [8, 35].

The main objective of this work is to minimize the YE requirement in d-lactic production by means of B vitamin addition and improve the fermentation efficiency through MICF process. First, the influence of nine B vitamins on cell growth and d-lactic acid fermentation was investigated to screen the essential B vitamins for *Lactobacillus delbrueckii*. Then, the optimal amount of essential B vitamins and trace element and the influence of fermentation parameters such as dilution rate were examined. Finally, the membrane-integrated continuous fermentation using rice straw hydrolysate as substrate was conducted under well-optimized nutrient and other experimental conditions to evaluate the effectiveness of suggested fermentation strategy on YE reduction and the improvement of d-lactic acid fermentation efficiency.

## Results and discussion

### Preliminary experiment: the influence of different nutrient supplementation on d-lactic acid production

The effects of supplementation with different nutrient sources (YE, meat extract (ME), peptone (PEP) and corn steep liquor (CSL)) on d-lactic acid fermentation by *L. delbrueckii* were investigated in model fermentation broth. The amount of each nutrient source added was equivalent to a nitrogen dose of 10 g/l YE (corresponding to 0.1% nitrogen). The cell mass, glucose consumption and d-lactic acid production after 18 h fermentation were measured for comparison (Additional file 1: Figure S1). In the case of YE and ME was supplemented, glucose was completely consumed after 15 h and 18 h cultivation, while 12.3 and 26.7 g/l glucose was left when PEP and CSL was used. The highest cell mass of 5.53 g/l and d-lactic acid concentration of 45.3 g/l was yielded by YE, confirming that YE was the most appropriate supplement for d-lactate production using *L. delbrueckii*.

**Fig. 1:**
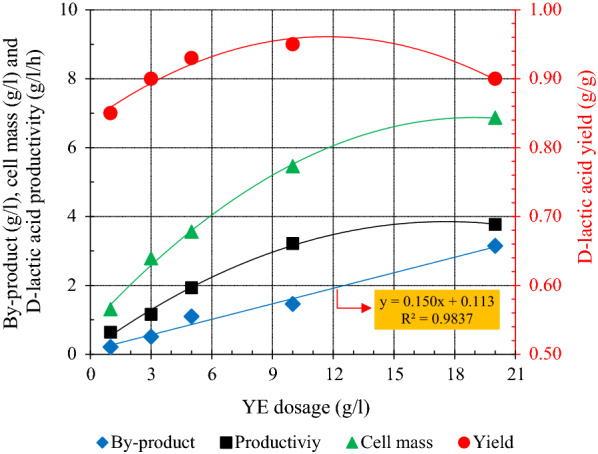
The relationships of D-lactic acid productivity, cell density and byproduct with various yeast extract doses in batch fermentation

To determine the optimum YE dose for fermentation, 1, 3, 5, 10 and 20 g/l YE was individually added to the model medium (abbreviated YE1, YE3, YE5, YE10 and YE20), and the influence of YE amount on cell mass, d-lactic acid productivity and yield and byproducts formation was depicted in Fig. 1. A linear relationship between byproducts (acetic acid and ethanol) and YE dose was observed. The byproducts increased from 0.21 to 3.75 g/l as the initial YE dose varied from 1 to 20 g/l. However, the productivity, yield and cell mass changed logarithmically, the maximum d-lactic acid yield of 0.95 g/g did not increase at dose higher than YE10, and more YE yielded no obvious enhancement in cell mass and productivity. The observations indicated that high level of YE addition induced higher titer byproducts, which led to inhibition of cell growth and fermentative ability. On the other hand, excess byproducts might cause the additional cost of product recovery and downstream processing. Therefore, the optimal dose of YE for D-lactic acid fermentation by *L. delbrueckii* was 10 g/l. However, to achieve the goal of low cost D- lactic acid production, YE addition should be reduced to a certain level. The observations implied that YE3 was the minimum amount for cell growth and fermentation. Besides, the byproducts titer was low and lactate yield was over 0.90 g/g. Therefore, YE3 was chosen to prepare the poor nutrient synthetic fermentation medium for the subsequent experiments.

### Screening essential B vitamin for d-lactic acid production

**Fig. 2:**
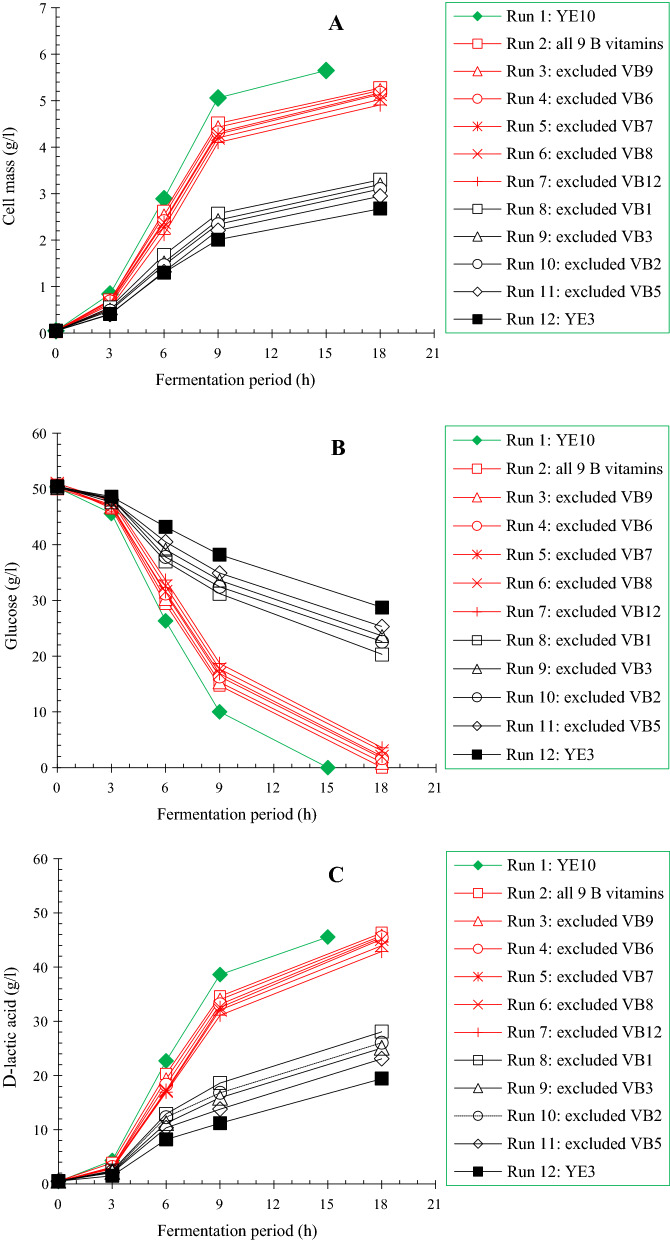
Screening of essential B vitamins for cell growth and D-lactic acid fermentation of *L. delbrueckii*. **A** Cell growth; **B** glucose consumption; **C** D-lactic acid production

B vitamins are integral nutrient for LAB growth and lactate fermentation. To understand the influence of individual B vitamins on d-lactic acid production by *L. delbrueckii*, screening experiments were conducted by means of excluding single B vitamins from the synthetic fermentation medium containing YE3. Nine B vitamins presented in YE were selected, and the individual supplementation amount was equivalent to that of YE10. Moreover, fermentation in medium comprising YE3, YE10 and YE3 coupled with full B vitamins was performed as a control. As Fig. 2 defined, the maximum and minimum cell mass and d-lactic acid titer was obtained from the control medium of YE10 and YE3, respectively. By supplementing full B vitamins in YE3 medium, the cell mass and d-lactate titer were significantly improved to levels close to those of YE10, implying the combined supplementation of YE3 and B vitamins could fulfill the nutritional requirement of *L. delbrueckii*, consequently overcoming the inhibition of cell growth and fermentation capacities caused by YE reduction, and maintaining the cell mass and d-lactic acid production at a high level. The elimination of vitamins B_6_, B_7_, B_8_, B_9_ and B_12_ (VB_6_, VB_7_, VB_8_, VB_9_ and VB_12_) gave no obvious influence on cell growth and fermentation abilities. The initial glucose of 50.6 g/l was almost consumed after 18 h fermentation with the maximum cell mass of 5.23, 5.17, 5.14 and 5.03 g/l and d-lactic acid titer of 45.5, 45.1, 43.9 and 42.8 g/l, close to that of control medium supplemented with YE3 and full B vitamins (5.27 and 46.3 g/l). In contrast in the case of VB_1_, VB_2_, VB_3_ and VB_5_ absence, the cell growth was similar to that of control medium with YE3, presenting a significant inhibitory profile. Accordingly, glucose consumption was severely delayed, over 20 g/l glucose left after 18 h fermentation, leading to a considerable reduction in d-lactic acid titers (28.1, 25.3, 26.0 and 23.1 g/l). The observations revealed that VB_1_, VB_2_, VB_3_ and VB_5_ were essential for *L. delbrueckii* growth and fermentation, which coincided with the published report showing that the four B vitamins played important roles in the regulatory mechanisms governing LAB growth and fermentation abilities. Klotz et al. [36] reported that VB_5_ was a component of coenzyme A (CoA) and acyl-carrier-protein (CAP) involved in protein and fatty acid synthesis of LAB. VB_2_ and VB_3_ were the precursors of cofactors such as FAD and NAD. NADH directly impacts lactic acid production as a cofactor of lactate dehydrogenase (LDH) [36, 37]. In addition, some studies figured out that B vitamin supplementation enhanced the activities of key enzymes of LA biosynthetic pathway in terms of phosphofructokinase (PFK) and L(+)-LDH. VB_1_ promoted glycolysis rate while VB_2_ accelerated electron transfer, thus increasing the metabolic flux of EMP pathway [37, 38].

### Effect of trace elements on *L. delbrueckii*d-lactic acid production

It is already known that trace elements have positive effect on lactate fermentation by promoting the cell growth of LAB and regulating the activities of the key enzymes involved in metabolism [39]. To elucidate the influence of trace elements in terms of Mn^2+^, Mg^2+^, P^3+^ and Fe^3+^ on *L. delbrueckii*, the individual constituents were excluded one-by-one in synthetic medium with four B vitamin and YE3 addition. d-Lactate accumulation and optical purity had no fluctuations in the absence of Mn^2+^, P^3+^ and Fe^3+^, but significantly decreased in the absence of Mg^2+^, indicating that Mg^2+^ was necessary for d-lactate production by *L. delbrueckii* (Table 1). Moreover, the optimum Mg^2+^ concentration for fermentation was also studied. The maximum d-lactate titer of 47.9 g/l and optical purity of 99.5% were achieved under Mg^2+^ concentration of 38.5 mg/l. Nevertheless, the relatively high dose of Mg^2+^ decreased the lactate titer, which might be contributed to the inhibitory influence on cell growth.

**Table1:** Effect of trace elements on d-lactic acid production by *L. delbrueckii*

Run	Medium			d-Lactic acid production	
	N source (g/l)	B vitamin supplementation^a^	Elements supplementation	Titer (g/l)	Optical purity (%)
13	YE (3.0)	+	Manganese, Iron, Magnesium	46.9	> 99.0
14	+	+	Phosphorus, Iron, Magnesium	47.3	> 99.0
15	+	+	Phosphorus, Manganese, Magnesium	47.1	> 99.0
16	+	+	Phosphorus, Manganese, Iron	1.7	97.2
			Magnesium (MgSO_4_·7H_2_O (g/l))		
17	+	+	0.2 (19.3)^b^	3.2	97.1
18	+	+	0.4 (38.5)	47.9	99.5
19	+	+	0.6 (57.8)	47.2	99.2
20	+	+	0.8 (77.0)	46.6	98.7
21	+	+	1.0 (96.3)	45.2	97.9

^a^ B vitamins was supplemented as follows (μg/l): thiamine·HCl (VB_1_), 1070; riboflavin (VB_2_), 495; niacin (VB_3_), 9840; Ca-pantothenate (VB_5_), 2370

^b^ Values in parentheses indicated the concentration of total magnesium content calculated using mg/l as unit

### Optimization of B vitamin supplementation in biomass hydrolysate

**Fig. 3:**
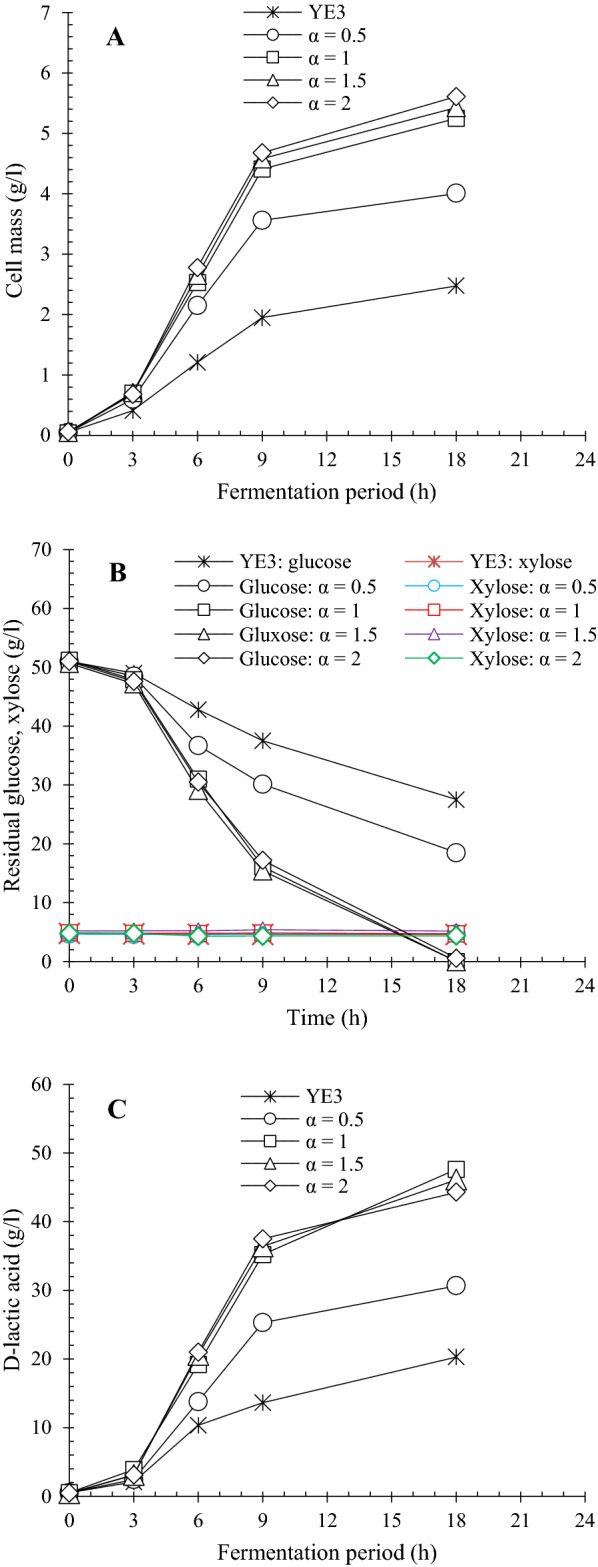
Effect of B vitamin dose on D-lactic acid fermentation by *L. delbrueckii.* **A** Cell growth; **B** Sugar consumption; **C** D-lactic acid production. Four essential B vitamins were supplemented in RSH medium with 3 g/l YE at enrichment factors of α = 0.5, 1, 1.5 and 2, respectively. B vitamin enrichment factor of α = 1: thiamine·HCl (VB_1_), 1070 μg/l; riboflavin (VB_2_), 495 μg/l; niacin (VB_3_), 9840 μg/l; Ca-pantothenate (VB_5_), 2370 μg/l

To determine the optimal dose of four B vitamins for D-lactic acid production by *L. delbrueckii*, fermentations were carried out in RSH medium with YE3 and varied enrichment factors of B vitamins (*α* = 0.5, 1.0, 1.5 and 2.0), respectively (Fig. 3 and Table 2). Fermentation in YE3 medium (*α* = 0) was conducted as the performance reference. The results showed that the increasing amount of B vitamins continuously stimulated the growth of *L. delbrueckii*, finally reaching the maximum cell mass of 5.61 g/l at *α* 2.0. Glucose was unable to be completely converted into lactate at α 0.5 after 18 h fermentation, resulting in a poor concentration of 30.7 g/l. When B vitamin addition was improved to α 1.0, the glucose was fully depleted, achieving the maximum lactate titer, productivity, yield and optical purity of 47.6 g/l, 2.64 g/l/h, 0.95 g/g and 99.5%, respectively. The lactate concentration was enhanced by 134.5% in comparison to that of control medium. Nevertheless, additional B vitamin supplementation led to slight decrease in d-lactate concentration and optical purity. Singhvi et al. [40] reported that lactate dehydrogenase expression in lactic acid producing bacteria was influenced by nitrogen sources, resulting in the enhancement of d-lactic acid production. Therefore, it was speculated that the increased supplementation of B vitamin in medium might cause the variation of lactate dehydrogenase involved in the isomerization reaction, consequently improving the amount of l-lactic acid during fermentation. However, the hypothesis needed further investigation. In conclusion, α 1.0 was considered as the optimal amount of B vitamin for the subsequent experiments.

**Table 2:** Effect of B vitamin dose on d-lactic acid production by *L. delbrueckii*

B vitamins (*α* =)^a^	Cell mass (g/l)	d-lactic acid				
		Titer (g/l/)	Enhancement by B vitamins (%)^b^	Productivity (g/l/h)	Yield (g/g)^c^	Optical purity (%)
0	2.48	20.3	-	1.13	0.86	98.3
0.5	3.98	30.7	51.23	1.82	0.90	98.8
1.0	5.21	47.6	134.48	2.64	0.95	99.5
1.5	5.43	46.2	127.56	2.57	0.93	99.2
2.0	5.61	44.3	118.23	2.46	0.89	97.6

^a^ α: enrichment factor of B vitamin. B vitamin (*α* = 1) was supplemented as follows (μg/l): thiamine·HCl (VB_1_), 1070; riboflavin

(VB_2_), 495; niacin (VB_3_), 9840; Ca-pantothenate (VB_5_), 2370

^b^ Enhancement (%) = [D-lactic acid titer (*α* = 0.5, 0.5, 1, 2)—D-lactic acid titer (*α* = 0)]/ D-lactic acid titer (*α* = 0)

^c^ D-lactic acid yield based on sugar consumed

### Membrane integrated continuous fermentation

**Fig. 4:**
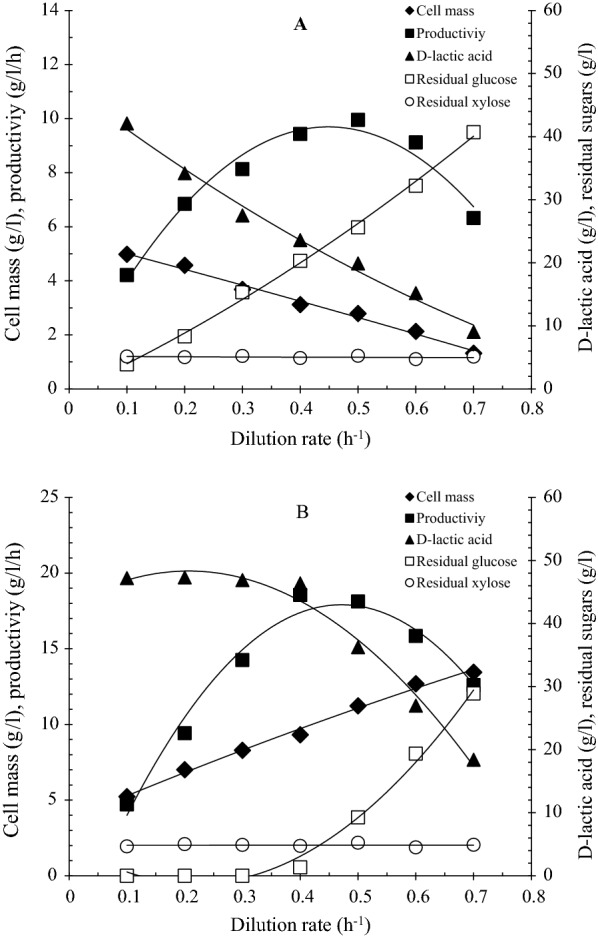
Effect of dilution rates (*D*) on D-lactic acid production during continuous fermentation with or without cell recycling by *L. delbrueckii*. **A** Conventional continuous fermentation system (without cell-recycle); **B** Cell-recycle continuous fermentation system

Continuous fermentation with and without cell recycling was conducted by RSH using four B vitamin together with YE3 as nutrient sources. To assess the optimal dilution rate for fermentation, the experiments were performed under dilution rates range of 0.1 h^−1^ to 0.7 h^−1^. The concentration of cell mass, residual sugar, d-lactic acid and productivity as a function of dilution rate were depicted in Fig. 4. In the case of MICF system (Fig. 4B), the initial glucose was thoroughly converted to d-lactic acid under the dilution rate 0.3 h^−1^, reaching d-lactate titer of 46.9 g/l. The cell mass progressively increased with the change of dilution rate and the cell density of 13.45 g/l was attained at the dilution rate of 0.7 h^−1^.The maximum productivity of 18.61 g/l/h was achieved when the dilution rate was set at 0.4 h^−1^, and then decreased with the increased dilution rate. In the case of fermentation without cell-recycle, the cell mass presented a liner decreasing trend with the variation of dilution rate, suggesting serious cell loss during fermentation (Fig. 4A). Accordingly, more residual sugars and a decrease in lactate concentration were observed. The maximum cell density and productivity were 4.98 g/l and 9.43 g/l/h, which were almost half of those obtained from MICF system. The observations indicated that the specific growth rate of cells was equal to the dilution rate during the lactate fermentation with cell recycling. In comparison to a free cell continuous fermentation, the membrane integrated fermentation effectively prevented cell loss via medium exchange and maintained cells growth at its maximum rate, thereby greatly enhancing the d-lactic acid concentration and productivity. Moreover, although the increasing dilution rate promoted the progressive cell growth due to the rapid delivery of fresh medium, but the more unconsumed sugar detected in fermenter because the fermentation broth had insufficient time to ferment. Overall, taking account the few residual sugars, high cell density and d-lactic acid concentration and productivity, a dilution rate of 0.4 h^−1^ was considered applicable for subsequent experiments.

In addition, the optimal nutrient conditions determined in batch fermentation were proven to be suitable for MICF system using RSH as substrate. With the supplementation of YE3 and four B vitamin, the d-lactic acid concentration attained at dilution rate of 0.4 h^−1^ was 46.4 g/l, slightly higher than that obtained in synthetic medium with YE10 (45.5 g/l) under batch fermentation mode.

**Fig. 5:**
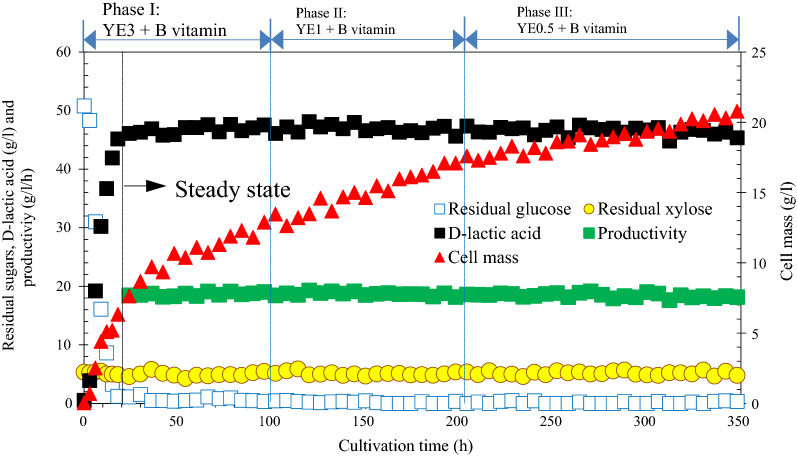
A 350 h membrane integrated continuous fermentation by *L. delbrueckii.* Phase I: 0–100 h (YE3); Phase II: 100–200 h (YE1); Phase III: 200–350 h (YE0.5). The dilution rate (*D*) for continuous operation was set at 0.4 h^−1^

A 350 h continuous fermentation with cell recycling was conducted by gradually reducing YE from initial 3.0 g/l (phase I) to 0.5 g/l (phase III). As Fig. 5 exhibited, the cell mass, d-lactate concentration and productivity presented a sharp increase during the first 18 h, then d-lactate production and productivity entered a steady state while the cell mass increased until fermentation was completed. Reducing the YE content in mixed nutrient source at different phases gave no influence on cell growth and lactate accumulation, the average d-lactate titer and productivity obtained under the three phases were almost identical, at approximately 46.6 g/l and 18.56 g/l/h, respectively. Such stable fermentation performance further demonstrated the positive joint effect of B vitamin supplementation and MICF on YE reduction and improvement in fermentation efficiency. The continuous fermentation with cell recycling under appropriate dilution rate could greatly reduce the cell washout and maintain the high cell density by B vitamin supplementation during the long term operation, thereby, the constant lactate titer and productivity could be achieved even with the continuous reduction of YE. In addition, no signs membrane fouling was observed in our fermentation system. The concentration of main byproducts in terms of acetic acid and ethanol examined after fermentation was < 0.7 g/l. The low concentration of byproducts led to a high chemical purity of d-lactate.

Table 3 summarized the recently published works on microbial d-lactic acid production from lignocellulosic wastes. Except for this study, all other reports were conducted in batch or fed-batch fermentation mode with different microbes. Regarding the nutrient sources, some agricultural wastes such as barley extract, whey protein hydrolysate, ME, soybean meal, cottonseed meal were used as single nutrients or supplemented with YE or YE and PEP. It was notable that the fermentation efficiencies in these studies were unsatisfied. The highest productivity value of 2.80 g/l/h was reported in the literature of fermentation from pulp mill residue using a mixed nutrient, which was far lower than that of our study of 18.56 g/l/h. Although various studies had been performed using MICF to improve l-lactic acid productivity, its application for d-lactic acid fermentation from lignocellulosic biomass was little. Hence, our experimental results provided important reference information for developing an economically efficient process for d-lactic acid production. Furthermore, the enantiomeric purity of d-lactate obtained in our work was 99.5%, which was preferred since high enantiomeric purities of more than 99.0% were needed to form heat-resistant PLA.

**Table 3:** Summaries of recently published works on microbial d-lactic acid production from lignocellulosic wastes using different nutrient sources

Substrate		Microorganism	Fermentation mode	D-lactic acid				References
Carbon sources	Nitrogen sources			Titer (g/l)	Productivity (g/l/h)	Yield ^a^(g/g)	O.P ^b^ (%)	
Bassage cellulose	YE	*L. lactis* mutant RM2-24	SSF (Batch)	73.0	1.52	0.73	–	[41]
Microalga	YE, PEP	*L. coryniformis* subsp*. torquens* ATCC 25,600	SSCF (Batch)	36.6	1.02	0.46	99.6	[42]
Curcuma Longa	SM	*L. coryniformis* ATCC 25,600	SSCF (Batch)	91.61	2.08	0.65	99.5	[43]
Pulp	YE, PEP, BE	*L. delbrueckii* ATCC 9649	SHF (Batch)	36.3	1.01	0.83	99.8	[44]
Hard wood pulp	BE	*L. plantarum* NCIMB 8826 (*ΔldhL1::PxylAB-Δxpk1::tkt-Δxpk2::PxylAB)*	SSF (Batch)	102.3	2.29	0.88	99.2	[22]
Sorghum stalks	YE, PEP	*L. plantarum* NCIMB 8826 (*ΔldhL1-*pCU*-*P*xylAB*)	SSCF (Batch)	22.0	0.65	0.55	99.0	[13]
Corn stover	YE, PEP	*L. plantarum* NCIMB 8826 (*ΔldhL1-*pCU*-*P*xylAB*)	SSCF (Batch)	27.3	0.75	0.68	99.0	[13]
Corn stover	SM	*L. plantarum* NCIMB 8826(*ΔldhL1-*pLEM*-xylAB*)	SSF (Fed-batch)	61.4	0.32	0.77	> 99.0	[29]
Corn stover	YE, PEP, BE	*P. acidilactici* ZP26 (*ldh* deficient strain)	SSF (Batch)	77.8	1.02	0.58^b^	99.3	[15]
Corncob residues	CM	*S. inulinus* YBS1–5	SHF (Fed-batch)	107.2	1.19	0.85	99.2	[20]
Pulp mill residue	YE, PEP, BE	*L. coryniformis* subsp*. torquens* ATCC® 25600™	SHF (Batch)	57.0	2.80	0.97	98.3	[45]
Wheat bran	WBH	*S. inulinus* YBS1–5	SSF (Fed-batch)	87.3	0.81	0.65	99.1	[46]
Corn stover	YE, PEP, BE	*L. delbrueckii* sp*. bulgaricus* CICC21101	SSF (Batch)	18.0	0.36	0.18^b^	99.0	[47]
Corn stover	YE, PEP, BE	*P. acidilactici* ZY15	SSCF (Batch)	97.3	1.01	0.65^b^	99.2	[10]
Corn stover	YE, PEP, BE	*P. acidilactici* ZY15	SSCF (Batch)	124.8	1.73	0.81^b^	> 99.0	[48]
Beechwood	YE, PEP, BE	*Lactobacillus delbrueckii* subsp*. bulgaricus* ATCC® 11842	SSF (Batch)	62.1	0.86	0.69	–	[49]
Corn stover	YE, PEP, BE	*P. acidilactici* ZY15-*ΔackA2::CGS9114_RS09725*	SSCF (Batch)	115.0	1.60	0.61^b^	> 99.0	[50]
Rice straw	YE, VB	*L. delbrueckii* subsp*. delbrueckii NBRC* 3202	MICF (Continuous)	46.6	18.56	0.92	99.5	This study

BE: Barley extract; CM: cottonseed meal; ME: meat extract; MICF: Membrane integrated continuous fermentation; PEP: peptone; SHF: Separate hydrolysis and fermentation; SM: soybean meal; SSCF: Simultaneous saccharification and co-fermentation; SSF: Simultaneous saccharification and fermentation; VB: Vitamins B; WBH: wheat bran hydrolysate; WPH: whey protein hydrolysate; YE: yeast extract

^a^ Yield refers to initial substrate amount

^b^ Optical purity of D-lactic acid

Finally, the YE content in our mixed nutrient source was 3.0 g/l in batch fermentation and an average of 1.5 g/l in continuous fermentation, far less than other studies, in which at least 5.0 g/l YE was required. Additionally, the supplementation amount of four B vitamins was minor, 13.8 mg/l in total. The unit prices of YE, VB_1_, VB_2_, VB_3_ and VB_5_ were $3.5, $36.6, $10.9, $9.1 and $9.3 per kilogram (≥ 99% purity, www.1688.com, China). Under batch fermentation, when YE10 was used as nutrient source, 222 kg YE was necessary to produce one ton of d-lactic acid product, adding $777.0 in cost. By substitution of YE10 with YE3 and four B vitamins, the YE cost could be reduced to $233.3; together with the cost of $3.5 of four B vitamins, the total nutrient cost could become $236.8, yielding a saving of 69.5%. During 350 h continuous fermentation with a membrane, the YE content was further reduced to 1.0 g/l and 0.5 g/l at phase II and phase III, respectively, which resulted in nutrient cost of $109.0, saving up to 86.0%.The results clearly defined the suggested strategy of four B vitamins supplementation combined with MICF system could significantly reduce the nutrient cost in fermentation by *L. delbrueckii* while also greatly improving the fermentation efficiency.

In conclusion, the research of using inexpensive nutrient sources to substitute YE provided a new insight into economical D-lactic acid production from lignocellulosic feedstock. However, there were still challenges required to be addressed to improve the process. One of the focuses for future work was to develop an effective pretreatment method to obtain the inherent B vitamin in lignocellulosic feedstocks, which would reduce the use of commercial B vitamin and thus further reducing the nutrient cost. The other important aspect was the selection of new D-lactic acid producing bacterial strains with the characteristics of highly productive, substrate and lactic acid resistant as well as capable of consuming all kinds of carbon sources presented in cheap feedstocks. Additionally, the improvement of lactic acid purification process e.g. simplifying the operation steps and eliminating the generation of byproduct to achieve production cost reduction was also expected.

## Conclusion

The observations indicated that B vitamins were a satisfying partial substitute for YE in d-lactic acid production from RSH by *L. delbrueckii*. The specific supplementation of four B vitamins together with YE could fulfill the nutritional requirements of *L. delbrueckii*. And the adoption of MICF could further reduce the YE amount and greatly improve d-lactate productivity. Hence, the novel fermentation strategy we proposed would be a promising solution for reducing the total manufacturing costs, consequently accelerating the industrial-scale production.

## Materials and methods

### Materials

Rice straw (RS) was harvested from a local farm (Dalian, China). The dried RS was milled to ≤ 2 mm and stored in plastic bags for further pretreatment. A commercial enzyme cocktail (Cellic® CTec2), acquired from Novozyme Inc. (A/S, USA) was used in enzymatic hydrolysis process. YE purchased from Merck KGaA. Meat extract, peptone (casein) and B vitamins bought from Sigma.

### Pretreatment of rice straw hydrolysate (RSH)

#### Prewashing treatment and liquid hot water pretreatment (LHWP)

The prewashing treatment of the RS was conducted at 10:1 (w/w) water/solid ratio in reactor with agitation rate of 50 rpm for 40 min. After prewashing process, the RS was separated by vacuum filtration and stored at 4 °C for subsequent liquid hot water pretreatment (LHWP).

LHWP of the prewashed RS was conducted in a pilot-scale high-pressure chemical reactor. LHWP was carried out at 185 °C and 50 rpm for 25 min with a 1:10 (w/w) solid/liquid (deionized water) ratio. After pretreatment, the reactor was immediately cooled to below 80 °C. The water-insoluble solids (WIS) and liquid fraction (prehydrolyzate) was then separated by vacuum filtration. The WIS was stored at 4 °C for use in the following experimental runs.

#### Enzymatic hydrolysis (EH)

WIS (6 kg) was subjected to enzymatic hydrolysis by cellulase (Cellic® CTec2) at dose of 15 FPU/g dry WIS. EH was carried out at 50 °C, 200 rpm for 72 h with 15% (w/w) of WIS loading in 50 mM citrate buffer (pH 5.0). After EH, the liquid fraction was centrifuged at 8000*g* for10 min, followed by filtration (MF, 0.22 μm/UF, MWCO 100 kDa). Finally, a hybrid membrane filtration process was carried out to simultaneously detoxify and concentrate the RSH [51]. The prepared RSH contained 50.85 g/l glucose, 4.37 g/l xylose and 0.82 g/l arabinose, with no other inhibitors detected.

### Microorganism and growth conditions

*Lactobacillus delbrueckii* subsp *delbrueckii* (NBRC 3202) from the NITE Biological Resource Centre (NBRC, Japan) was used in this study. For inoculum, the cells were transferred to 100 ml sterile MRS broth then cultured at 40 °C with shaking rate of 130 rpm on a rotary shaker. After 24 h cultivation, the cells were harvested by centrifugation and then washed twice. An inoculation size of 10% (v/v) was used for fermentation.

### Fermentation

#### Batch fermentation

Batch fermentation runs were conducted at 40 °C, 300 rpm agitation speed and N_2_ gas was introduced into the a BIOSTAT® B-plus 2-l bioreactor (Sartorius, Germany) at 0.2 l/min to maintain anaerobic environment with a starting working volume of 1 l. The pH was automatically regulated at 6.0 using ammonia during batch fermentation.

The model medium for fermentation contained the following basic ingredients (g/l): glucose, 50; sodium acetate, 1; K_2_HPO_4_, 0.5; KH_2_PO_4_, 0.5; MgSO_4_·7H_2_O, 0.4; FeSO_4_·5H_2_O, 0.03; MnSO_4_·5 H_2_O, 0.05; and various amounts of YE (1, 3, 5, 10 and 20 g/l YE, abbreviated YE1, YE3, YE5, YE10 and YE20) and B vitamins. The basal B vitamins were added to yield a fermentation broth based on the analytical data of YE10 [36]. B vitamins had the following composition: thiamine·HCl (VB_1_), 1070 μg/l; riboflavin (VB_2_), 495 μg/l; niacin (VB_3_), 9840 μg/l; Ca-pantothenate (VB_5_), 2370 μg/l; pyridoxine·HCl (VB_6_), 939 μg/l; biotin (VB_7_), 18.3 μg/l; inositol (VB_8_), 58.4 μg/l; folic acid (VB_9_), 583 μg/l; cyanocobalamin (VB1_2_), 0.93 μg/l.

YE3 was added to synthetic or RSH medium to prepare minimal media. To determine the optimal dose of B vitamins for cell growth and fermentation, four essential B vitamin were supplemented in minimal media with enrichment factors of *α* = 0.5, 1, 1.5 and 2, respectively. The enrichment factor of *α* = 1 defined as the amount of four B vitamins presented in 10 g/l YE (VB_1_, 1070 μg/l; VB_2_, 495 μg/l; VB_3_, 9840 μg/l; VB_5_, 2370 μg/l).

#### Continuous fermentation

**Fig. 6:**
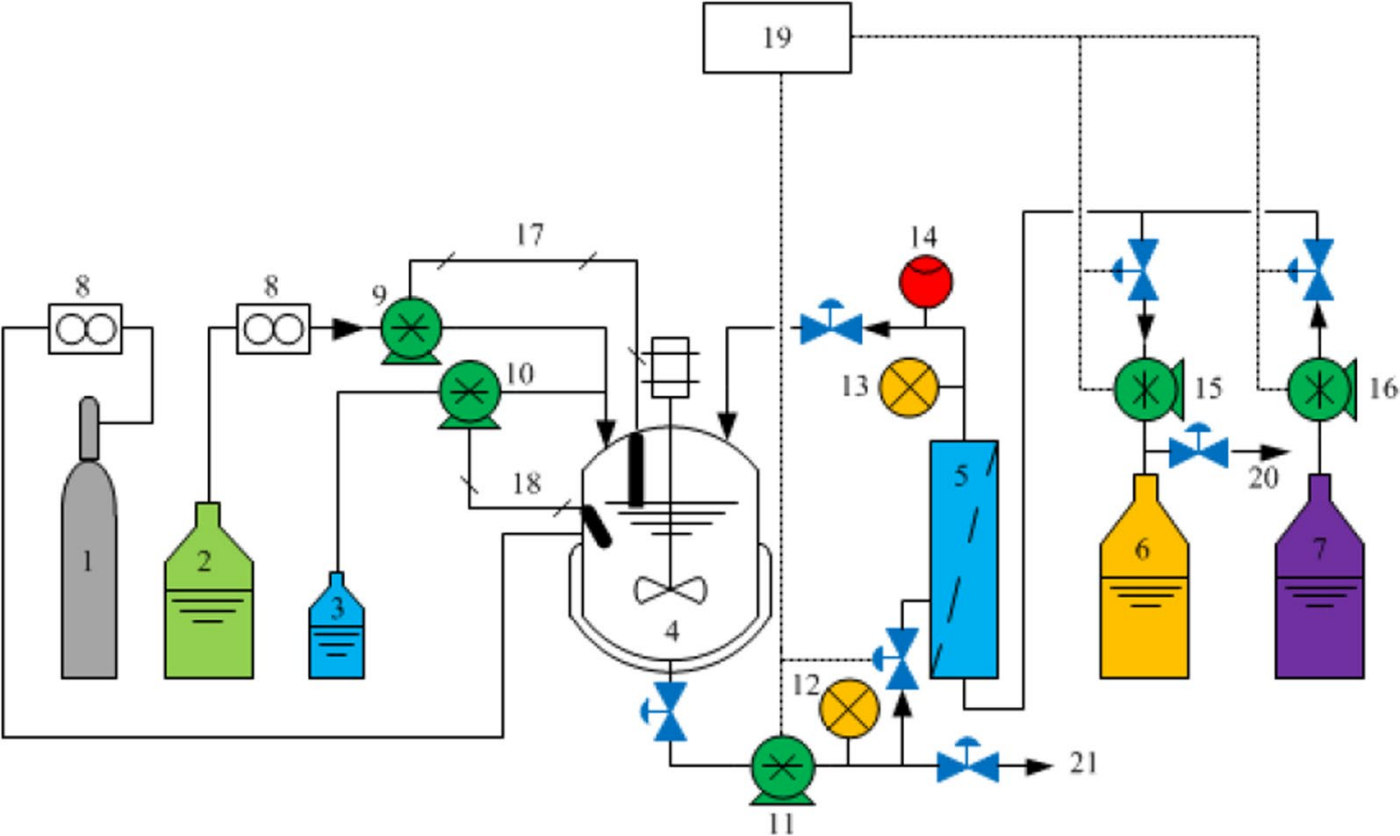
Schematic diagram of the membrane integrated continuous fermentation system. (1) N_2_ storage tank; (2) Feed medium storage tank; (3) Neutralizer reservoir; (4) Fermentor; (5) Hollow-fiber microfiltration module; (6) Product storage tank; (7) Cleaning solution tank; (8) Flow meter; (9) Medium feed control pump; (10) Alkali feed control pump; (11) Recirculation pump; (12) Feed pressure indicator; (13) Retentate pressure indictor; (14) Retentate flow rate indicator; (15) Permeate control pump; (16) Backwash pump; (17) Level sensor; (18) pH sensor; (19) Control host; (20) Sampling (analysis of residual sugars and d-lactic acid); (21) Sampling (analysis of cell growth)

Continuous fermentation was performed in a BIOSTAT® B-plus 2-l bioreactor (Sartorius, Germany) coupled with a hollow-fiber microfiltration module (MICROZA PSP 103, 42 mm × 347 mm; Asahi Kasei, Tokyo, Japan) for cell recycling. Figure 6 presents a schematic diagram of the membrane-integrated continuous fermentation (MICF) system. The module composed polyethylene hollow-fibers (ID, 0.7 mm) with a pore diameter of 0.1 μm and an effective surface area of 0.17 m^2^. The fermentation changed from initial batch mode to continuous mode after complete consumption of glucose with the agitation speed increased from 300 to 800 rpm at 40 °C with N_2_-flow rate of 0.2 l/min. The broth was sustained at 1 l by supplying fresh RSH containing approximately 55 g/l reducing sugar, 38.5 mg/l Mg^2+^, 3 g/l YE and four essential B vitamins with enrichment factor of *α* = 1.0. The influence of dilution rate (*D*) on fermentation was examined in the range of 0.1 h^−1^ to 0.7 h^−1^. Sampling was performed five times under each dilution rate after fermentation reached steady state. Fermentation without cell-recycle was simultaneously performed as control.

### Analytical methods

Cell growth was measured periodically by monitoring the optical density (OD) at 660 nm using a spectrophotometer (UV-1600, Shimadzu, Japan) and calculating the dry cell weight (DCW) by an appropriate calibration curve. The sugars and metabolites were quantified using HPLC (Agilent Technologies 1200 Series, USA) equipped with a refractive index detector using an Aminex HPX-87H column (Bio-Rad, Richmond, CA, USA) as described previously [51]. The optical purity of D-lactic acid was assayed by HPLC using a chiral column (50 mm × 4.6 mm ID; MCI GEL CRS15W; Mitsubishi Chemical Co., Tokyo, Japan) at 254 nm. The optical purity of D-lactic acid was calculated with the following equation:

$$ {\text{Optical purity }}\left( \% \right) \, = { 1}00 \times \, \left( {{\text{D}} - {\text{lactic acid titer }} - {\text{ L}} - {\text{lactic acid titer}}} \right)/({\text{D}} - {\text{lactic acid titer }} + {\text{ L}} - {\text{lactic acid titer}}). $$

## Supplementary Information

**Additional file 1: Figure S1.** Effect of various nitrogen sources on D-lactic acid production. The amount of each nutrient source added was equivalent to a nitrogen dose of 10 g/l YE (corresponding to 0.1% nitrogen). The concentration of nitrogen sources added (g/l): yeast extract (YE), 10; meat extract (ME), 8.4; corn steep liquor (CSL), 14.2; peptone (PEP), 7.7.

## Abbreviations

BE: Barley extract
CM: Cottonseed meal
CSL: Corn steep liquor
D: Dilution rate
DCW: Dry cell weight
EH: Enzymatic hydrolysis
LAB: Lactic acid-producing bacteria
LHWP: Liquid hot water pretreatment
ME: Meat extract
MICF: Membrane integrated continuous fermentation
PEP: Peptone
RSH: Rice straw hydrolysate
SH: Soybean hydrolysate
SHF: Separate hydrolysis and fermentation
SM: Soybean meal
SSCF: Simultaneous saccharification and co-fermentation
SSF: Simultaneous saccharification and fermentation
VB: Vitamins B
WBH: Wheat bran hydrolysate
WIS: Water-insoluble solids
WPH: Whey protein hydrolysate
YE: Yeast extract
