# Supplementary material for: d-Lactic acid production from agricultural residues by membrane integrated continuous fermentation coupled with B vitamin supplementation
Source: Biotechnol Biofuels Bioprod. 2022 Mar 4;15:24. doi: 10.1186/s13068-022-02124-y (PMC8897852; doi:10.1186/s13068-022-02124-y)
Supplement: Supplementary file 1 — Additional file 1: Figure S1. Effect of various nitrogen sources on D-lactic acid production. The amount of each nutrient source added was equivalent to a nitrogen dose of 10 g/l YE (corresponding to 0.1% nitrogen). The concentration of nitrogen sources added (g/l): yeast extract (YE), 10; meat extract (ME), 8.4; corn steep liquor (CSL), 14.2; peptone (PEP), 7.7. [file 13068_2022_2124_MOESM1_ESM.docx]

**Supporting information Figure Captions:**

Figure S1. Effect of various nitrogen sources on D-lactic acid production. The amount of each nutrient source added was equivalent to a nitrogen dose of 10 g/l yeast extract (corresponding to 0.1% nitrogen). The concentration of nitrogen sources added (g/l): yeast extract (YE), 10; meat extract (ME), 8.4; corn steep liquor (CSL), 14.2; peptone (PEP), 7.7.

Figure S1
